# Effect of authentic leadership and mindfulness educational program on nursing managers' competencies: a quasi-experimental study

**DOI:** 10.1186/s12912-024-01976-z

**Published:** 2024-05-21

**Authors:** Warda Mohammed Elsayed Shurab, Sanaa Abd Elazim Ibrahim, Marwa Mohamed Abdelaalem, Samar Atiya Abosaleh Gabal, Takwa Rashwan Mohamed Abdelhady

**Affiliations:** Faculty of Nursing, Port Said University, Port Said, Egypt

**Keywords:** Authentic, Competencies, Leadership, Mindfulness, Nursing management, Nurse managers

## Abstract

**Background:**

Authentic leadership is an emerging perspective in leadership that focuses on leaders' values and beliefs. while the mindfulness perspective permits nurse managers to be fully present, aware of themselves and their impact on others, and aware of their reactions in stressful situations. so, authentic leadership and mindfulness if combined create nurse managers who have clearer, more focused thinking, and a growth mindset that help subordinates improve and grow. as well as mindfulness-based interventions help them to improve interpersonal relationships with patients and colleagues, and to take better care of themselves and others.

**Aim:**

The present study aims to; explore the effect of authentic leadership and mindfulness educational program on nursing managers' competencies in hospital.

**Methods:**

A quasi-experimental design (Quantitative pre-, post-, and follow-up design) was used to conduct the study at Shirbeen General Hospital, Egypt. The study subjects consist of a purposive sample of 70 nurse managers and 226 nurses. Three tools used for data collection consisted of; the authentic leadership questionnaire, the five Facet Mindfulness Questionnaire, and the managerial competencies of Nurse Managers. Data analysis was performed using SPSS version 20, Qualitative categorical variables were compared using the chi-square test. A significant level value was considered when the *p*-value ≤ 0.05, and Cohen's d was used to measure the effect size which indicated there was a large effect of educational program on post and follow-up knowledge, authentic leadership, mindfulness, and managerial competencies scores.

**Results:**

The current study revealed that there were significant differences between nurse managers’ knowledge, authentic leadership, mindfulness, and managerial competencies (*P* = 0.001) pre-, post-, and after 3 months of the program. As determined by Cohen’s d test, there was a large effect of educational program on post and follow-up knowledge, authentic leadership, mindfulness, and managerial competencies scores.

**Conclusion:**

The educational program about authentic leadership and mindfulness had a positive effect on nurse managers’ managerial competencies.

**Trial registration number (TRN):**

The study protocol was approved by the Research Ethics Committee of the Faculty of Nursing, Port Said University, Egypt (code number: NUR 13/3/2022–11).

## Background

Authentic leadership (AL) has become one of the most important leadership approaches in light of recent societal changes globally. Modern organizations require leaders who: possess high ethical standards, are aware of their abilities, and are fair in their decision-making process by creating integrity in the workplace [1, 2]. Authentic leadership defined as a pattern of leader behavior that draws upon and promotes both positive psychological capacities and a positive ethical climate, to foster greater self-awareness, an internalized moral perspective, balanced processing of information, and relational transparency on the part of leaders working with subordinates, fostering positive self-development [3].

Zhang et al. [4] classified authentic leadership outcomes as: 1. Follower attitudinal outcomes: positively related to subordinates' psychological empowerment, work engagement, psychological capital, psychological safety, job autonomy, organizational commitment, and job satisfaction. On another hand, authentic leadership is negatively related to stress, emotional exhaustion, and cynicism [2]. 2. Follower behavioral outcomes: authentic leadership has an effect on two key work behaviors: increasing organizational citizenship behaviors (OCB) “which refers to individual behavior that is discretionary, not directly or explicitly recognized by the formal reward system”. And decreasing counterproductive work behaviors (CWB) “which refers to behavior that has a detrimental effect on organizations and their members”. 3. Leader-related outcomes: authentic leadership enhances the ability of leaders to perform their duties (leader effectiveness) and the quality of their exchange relationship with subordinates.

Authentic leaders are characterized by openness, integrity and have positive psychological traits such as trust, optimism and high morality. Additionally, authentic leaders effectively handle change and help employees cope [5]. On another hand, Frasier [6] describes authentic leaders as positive, ethical, values-driven, and collaborative, and in displaying these behaviors, they earn the trust and respect of their followers and influence follower performance.

Fry and Kriger [7] suggest that the greatest obstacle to experiencing reality authentic leadership is over-emphasis on the thinking mind, which creates an opaque screen of concepts, labels, judgments and definitions that blocks all true relationship, and that this over-emphasis decreases when attention is focused on the present. These characteristics suggest that mindfulness – which consists of putting aside personal filters to establish direct contact with experience and responding to that experience in a less-automatic, more-flexible way [8]. According to Nübold et al. [9] one factor that has been theorized to show a strong conceptual link to authentic leadership is mindfulness, and treat mindfulness as a personal antecedent to and a holistic means of training authentic leadership. Mindfulness promotes authenticity by allowing self-discovery and self-awareness, leading to more self-concordant goal setting.

Theoretically, Mindfulness-based interventions and authentic leadership are connected in that both emphasize self-awareness [10, 11]. Increased self-awareness of thoughts, emotions, and values developed through participating in Mindfulness-based interventions can foster a greater sense of autonomy in the leader, ultimately resulting in a more unified and self-determined, authentic sense of self [12, 13]. Pérez et al. [14] defined mindfulness as an approach to experiencing everyday life by directing attention and awareness to the present moment without judgment. Mindfulness is often associated with meditation, the practice of focusing on a chosen object of attention such as breath; however, the conceptualization of mindfulness practice extends beyond meditation or breathing techniques [15, 16].

According to Goestjahjanti et al. [3] the benefits achieved by practicing mindfulness leadership are: 1. self-awareness: knowing internal status, preferences, resources, and intuitions. 2. self-management: turning compulsion into a choice, managing, impulses, resources, and intuitions. 3. motivation: knowing what is important to self, aligning with leader values, cultivating resilience. 4. empathy: awareness of the feelings of others, cultivating connection and trust. 5. Social skills: cultivating communication skills especially listening, engaging skillfully with conflict, and leading with compassion.

As a leader, nursing managers (also called Head Nurses) are responsible for maintaining the link between an institution's administrative mission and the nurses who provide nursing care in the clinical unit, as well as being in charge of efficient patient care activities by ensuring that subordinate nurses are qualified for the tasks allocated to them. This is one of the reasons why their roles are considered the most complex in healthcare institutions [17]. To do their duties, they must promote the growth of mindset, adhere to a code of ethics, be open, transparent, and honest in their interactions, be genuine, and instill a work culture of personal growth, clarity, accountability, and innovation [18].

Competencies are attributes underpinning behavior, which at the individual level are a combination of knowledge, skills, and abilities where skills are outcomes of continuous learning and practice supported by a person’s abilities [19]. In the same context, managerial competence is described as a combination of knowledge, skills, abilities, and behaviors that an employee uses in an organization [20]. Ofei, Paarima, and Barnes [21] classified the managerial competencies as technical, conceptual, and human relationship competencies.

As a result of increased self-awareness, nurse managers reported success in transforming organizational culture, improvement in emotional intelligence, better managerial competencies, minimized psychological and physical impairment due to stress [16]. In this context, the current study aimed to examine the effect of implementing an educational program about authentic leadership and mindfulness on nurse managers' competencies, and determine if there was a positive relation between developing a mindful authentic leader and improving their technical, conceptual, and human relationship competencies.

### Significant of the study

Authentic leadership and mindfulness practices if combined together create head nurses that have clearer, extra focused thinking, and growth mindset that help subordinates improve and grow. Furthermore, the concept of authentic leadership style and mindfulness practice has strong impact on head nurses’ self-evaluation [18]. As well as, Ali et al. [22] asserted that authentic leadership educational program should be recognized and implemented for all head nurses and whom in a management position in the nursing field.

In Egypt, A study conducted by Elmawla et al. [14] found that the implementation of an educational program about authentic leadership and mindfulness increased head nurses’ authentic leadership to 65.7% post-program compared with pre-program implementation (50%) and Pre-program low percent 8.6% of the head nurse had a high level of total mindfulness level improved to be 98.6% immediately post program., also the program was effect positively on head nurses self-evaluation.

Another study by Aboelenein, and Mostafa [23] found that head nurses' authentic leadership style levels were low pre-program implementation (14.8%) while they had higher scores (85.2%) with statistically significant differences post-program implementation (*p*-value < 0.001), also found that staff nurses' resilience and innovation score were increased post-program implementation. Therefore, the present study was conducted to design and implement an educational program about authentic leadership and mindfulness practices and evaluate the effect of the educational program on nursing managers' managerial competencies.

### Study aim

The study aimed to explore the effect of authentic leadership and mindfulness educational program on nursing managers' competencies in hospital. And find the relation between authentic leadership, mindfulness, and managerial competencies of nurse managers.

### PICOT research question was:

In nurse managers what is the impact of authentic leadership and mindfulness educational program on their managerial competencies compared with pre-program within five months?

The study hypothesized that the educational program about authentic leadership and mindfulness will improve nursing managers' competencies.

## Methods

### Study design

A quasi-experimental study design was utilized to achieve the aim of this study [24]. Non-randomized comparative trial was used (comparison one group at pre-, post-, follow-up of the program) following the Guidelines for Reporting Non-Randomized Studies [25], and the Template for Intervention Description and Replication (TIDieR) checklist [26].

### Study setting

The present study was conducted at Shirbeen General Hospital which is affiliated to the Egyptian Ministry of Health—Dakahlia Governorate, Egypt. The hospital specializes in providing tertiary medical services for cases in Shirbeen City. The hospital’s capacity is 184 beds. The hospital includes 27 departments. The total nurse force is 714 nurses (204 B.Cs., 265 nursing technical institute, and 245 technical deplume 3 years).

### Study subjects

Consists of two groups, the first group consists of a purposive sample nurse manager. The study sample includes nurse managers working morning and afternoon shifts with a number of 70 from the total of (81 nurse managers). The study sample includes the nurse manager’s office, head nurses, assistant head nurse, charge nurse (alternative head nurse in only afternoon shifts), nurses’ supervisors, and hospital supervision teams (infection control team, quality team, training team, occupational health and safety team, and surveillance team) all are nurse specialists.

***Inclusion criteria of nurse managers***: Had at least one year of experience in their current position.

***Exclusion criteria of nurse managers: ***who worked only in the night shift.

The second group consists of (226) staff nurses from a total of (644 staff nurses) who work as subordinates for the studied nurse managers. The sample size of staff nurses was calculated according to the following Eq. [27]:

$$\frac{\mathrm{N }\times \mathrm{P }\left(1 -{\text{P}}\right)}{{\text{N}}-1\times \left({{\text{d}}}^{2}\div {z}^{2}\right)+\mathrm{P }\left(1-{\text{P}}\right)}$$

Where, *n* = sample size; N, studied total population; d = error percentage (= 0.05); P = prevalence or proportion of event of interest for the study; Zα/2 =1.96 (for 5% level of significance). Therefore,

$$n=\frac{644\times0.343\left(1-0.343\right)}{(644-1)\times\left(0.05^2\div1.96\right)+0.343\left(1-0.343\right)}=225.3$$

Accordingly, the sample size required is 226.

### Data collection tools

Three tools were used for data collection:

Tool (1): authentic leadership scale: That consisted of two parts as follow.

- Part (I): Authentic leadership knowledge questionnaire:

This part included self-reported questionnaire that was developed by the researcher into Arabic language, based on literature review as (Small, 2021; Wiewiora & Kowalkiewicz, 2019; Stedham & Skaar, 2019) to assess nurse managers' knowledge regarding authentic leadership before and after the program implementation. It consisted of 40 questions answered by (2 = yes, 1 = no, and 0 = I don’t know).

- Part (II): Self-reported questionnaire about authentic leadership:

This tool was adapted from Walumbwa et al. [28] to assess head nurses’ authentic leadership practice, it consists of a 16-items that measured four factors of authentic leadership: self-awareness four items, internalized moral perspective four items, balanced processing three items, and relational transparency five items. Items were rated on a five-point Likert scale (1- almost never, 2- never, 3- Sometimes, 4- always, 5- almost always). The estimated internal consistency alphas (Cronbach’s alpha) for each of the measures were also at acceptable levels: self-awareness, 0.79; relational transparency, 0.72; internalized moral perspective, 0.73; and balanced processing, 0.76 Walumbwa et al. [28]. Levels of authentic leadership were presented as: High level of authentic leadership > 64—80% = 52—64 scores, Moderate level of authentic leadership > 48—64% = 39—51 scores, Low level of authentic leadership > 32—48% = 26—38 scores.

### Tool 2: five facet mindfulness questionnaire (FFMQ)

The scale was developed by Baer et al. [29] and modified by the researcher to assess nurse managers’ mindfulness practice and translated into Arabic language by the researcher. The questionnaire consists of 39 items and was composed of five subscales: observing (8 items), describing (8 items), acting with awareness (8 items), non-judging of inner experience (8 items), and non-reactivity to inner experience (7 items). Alpha coefficients for Observe, Describe, Act with Awareness, and Accept Without Judgment were 0.91, 0.84, 0.83, and 0.87, respectively. Baer et al. [29]. Items were rated on a five-point metric of frequency (1- almost never, 2- never, 3- Sometimes, 4- always, 5- almost always). Levels of mindfulness were presented as: High level of mindfulness > 64—80% = 125—156 scores, Moderate level of mindfulness > 48—64% = 94—124 scores, Low level of mindfulness > 32—48% = 62—93 scores.

### Tool 3: The management competencies of nurse manager:

An adapted tool was developed by Ofei, Paarima, and Barnes [21] and was translated into Arabic language by the researcher. This tool consists of 27 items to assess nurse managers’ managerial competencies by their subordinates (staff nurses). Items were grouped according to the management competencies proposed by the Katz model (technical items, human relationship items, and conceptual items). Items were on a five-point Likert scale (1- not at all, 2- to a small extent, 3- to some extent, 4- to a large extent, and 5- to a very large extent). The questionnaire was validated through a pilot survey and expert advice, the overall Cronbach’s alpha of the questionnaire was 0.845 which is considered acceptable Ofei, et al. [21]. Levels of managerial competencies represented as: High level of managerial competencies > 64 - 80% = 100 - 125 scores, Moderate level of managerial competencies > 48 - 64% = 70 - 99 scores, Low level of managerial competencies >32 - 48 % = 48 - 69 scores.

*In addition to personal and work-related data*

It was developed by the researcher in Arabic language and included personnel and work characteristics of nurse managers such as age, gender, level of education, job title, marital status, years of experience, and previous attendance of an educational program in the scope of the study.

### Tool validity

The study tools were translated into the Arabic language by a language expert and then retranslated into the English language again before examining its validity. Content Validity of the translated tools was ascertained by a panel of experts consisting of 14 experts (seven experts from the nursing Administration department and seven experts from the psychiatric nursing department). Professors reviewed the three instruments for Arabic language translation clarity, relevance, comprehensiveness, and understanding applicability. The average proportion of Content Validity Index (CVI) for items judged relevant across the fourteen experts = 0.80. Comments and suggestions of the jury were considered and necessary modifications of items Arabic translation were done accordingly.

### Tool reliability

The reliability of tools used in this study by the Cronbach’s alpha coefficient test to assess the internal consistency of the study tools. The internal consistency reliability for the authentic leadership knowledge tool was (0.85), and for the authentic leadership questioner was (0.73). The internal consistency reliability for the five-facet mindfulness questionnaire was (0.96). While the overall Cronbach’s alpha of the management competencies of nurse managers was (0.85).

### Pilot study

Before entering the actual study, face validity was carried out by a pilot study on 10% (7 nurse managers and 26 nurses) of the sample to assess the clarity, practicability, and feasibility of the tool and to estimate the proper time required for the interview [30]. Appropriate modifications were made according to the results of the pilot study. Nurse managers and nurses who participated in the pilot study were excluded from the study subjects.

### Ethical considerations

The study was approved by the Research Ethics Committee (REC), Faculty of Nursing/ Port Said University with (code number: NUR 13/3/2022–11) based on the standard of the committee, Faculty of Nursing/ Por Said University. An official letter containing the title and the aim of the study was sent from the Dean of the Faculty of Nursing—Port Said University to the director of each setting to obtain approval from the hospital administrator for data collection in the abovementioned settings. Furthermore, written consent for participation in the study was obtained from nurse managers and staff nurses after clearing out all aspects of the study.

### Fieldwork

A field study was conducted for five months from the beginning of January (2023) to the end of May (2023). The study was carried out through the following phases:

- ***Phase I (Assessment phase):*** In this stage obtaining official permissions to carry out the study, the researcher visited the study settings and arranged with the nursing director for the actual implementation of the study. Then, the process of recruitment of nurse managers and their subordinates (staff nurses). Non-randomized comparative trial was used (comparison one group at pre-, post-, follow-up of the program) following the Guidelines for Reporting Non-Randomized Studies [25], and the Template for Intervention Description and Replication (TIDieR) checklist [26]. Participants who satisfied the inclusion criteria were invited to participate in this study and signed a written informed consent form. The researcher clarified the sheets of the three tools (knowledge questionnaire, authentic leadership questionnaire, and Five Facet Mindfulness Questionnaire) to each nurse manager and the fourth tool (managerial competencies questionnaire) to staff nurse and asked them to complete them before conducting the educational sessions. Each tool was filled in about 15 minutes to 30 minutes.
- ***Phase II (Planning):*** The educational program was designed based on the assessment data collected in Phase I. The educational program was aimed to enhance nursing managers' competencies regarding authentic leadership and their mindfulness practices. The educational program was designed to cover information that contributes to developing the studied nurse managers’ knowledge about authentic leadership and mindfulness practices. The handout includes theoretical content and procedures of fits caring was prepared to facilitate remembering knowledge about authentic leadership and mindfulness practices. The program covers the following two parts: the first part was about authentic leadership (definition, components, dimensions, and outcomes from implementing authentic leadership). The second part was about mindfulness (definition, purpose, importance, principles, dimensions, components, effect of mindfulness on leadership, characteristics of mindfulness leader, mindfulness-based training, and mindfulness exercises).
- ***Phase III (The educational program implementation***)**:** At the beginning, the researcher met with each nurse manager individually, explained the aim and procedures of the study, and invited them to participate. The participated nurse managers distributed into three groups each group include 23 nurse managers. The educational sessions conducted as two days per week for each group as the following table:

**Table:** 

Weeks Groups	1st week	2nd week	3rd week	4th week
**1**^**st**^**group (*n* = 23)**	Saturday	Saturday	Saturday	Saturday
	Tuesday	Tuesday	Tuesday	Tuesday
**2**^**nd**^**group (*n *= 23)**	Sunday	Sunday	Sunday	Sunday
	Wednesday	Wednesday	Wednesday	Wednesday
**3**^**rd**^** group (*n* = 24)**	Monday	Monday	Monday	Monday
	Thursday	Thursday	Thursday	Thursday

The training sessions were carried out by the researcher in the hospital's training class, each session lasted 60 minutes. A copy of the handout was given to each nurse manager to facilitate remembering the knowledge and practices during the explanations of the theoretical part. The program conducted by the Arabic language to avoid misunderstanding and presented in clear and concise form using different teaching methods while discussing with them the rationale and the precaution for each step as small discussions, lectures, demonstrations, and re-demonstrations and appropriate teaching media as audiovisual material and real objects. The nurse managers were introduced to the concepts and given practical training on authentic leadership behaviors and mindfulness exercises as (Body scan, Mindful eating, and Breath awareness). The training sessions also included guidance on incorporating mindfulness into daily life, emotion regulation strategies, and cultivating positive states of mind through self-awareness. Moreover, the educational program focused on deliberately reflecting on how the nurse managers could apply mindfulness approaches to their work.

At the end of the researcher's demonstrations nurse managers were asked about any unclear steps which needed repetitions or explanation before re-demonstration. The researcher emphasized that this session was done for teaching purposes not for evaluation, so mistakes and forgetting were allowed and were corrected immediately by the researcher.

- ***Phase IV* (*Evaluation phase*):** The program outcomes was evaluated by using the study’s three tools (knowledge questionnaire, authentic leadership questionnaire, and Five Facet Mindfulness questionnaire) immediately after program implementation and after three months of the program. As well as, the fourth tool (managerial competencies questionnaire) revaluated by staff nurses to assess the progress of nurse managers competencies post-program and after three months.

### Statistical analysis

The collected data were organized, tabulated, and statistically analyzed using SPSS for Windows version 20.0 (SPSS, Chicago, IL). The reliability (internal consistency) test for the questionnaires used in the study was calculated by Cronbach’s alpha coefficient test. Continuous data were normally distributed and are expressed as the mean ± standard deviation (SD). Categorical data are expressed numbers and percentages. The chi-square test (or Fisher’s exact test when applicable) was used for comparison of variables with categorical data. Pearson’s correlation (r) coefficient was adopted to analyze the correlations among key study variables. A significant level value was considered when the *p*-value ≤ 0.05. Cohen's d used to measure the effect size was considered small effect size at <0.5, medium effect size at 0.5<0.8, and large effect size at>0.8.

## Results

### Personal characteristics of the study subjects

Table 1 illustrates the personal characteristics of studied nurse managers, indicating that 68.6% of studied nurse managers were their age ranged from 35 to 40 years old. While 44.3% of them were single. Also, 41.4% of them had a diploma degree, and 61.4% had 10 to 15 years of experience. Furthermore, 52.9% of studied nurse managers were working as nurse managers for less than 5 years.

**Table 1:**
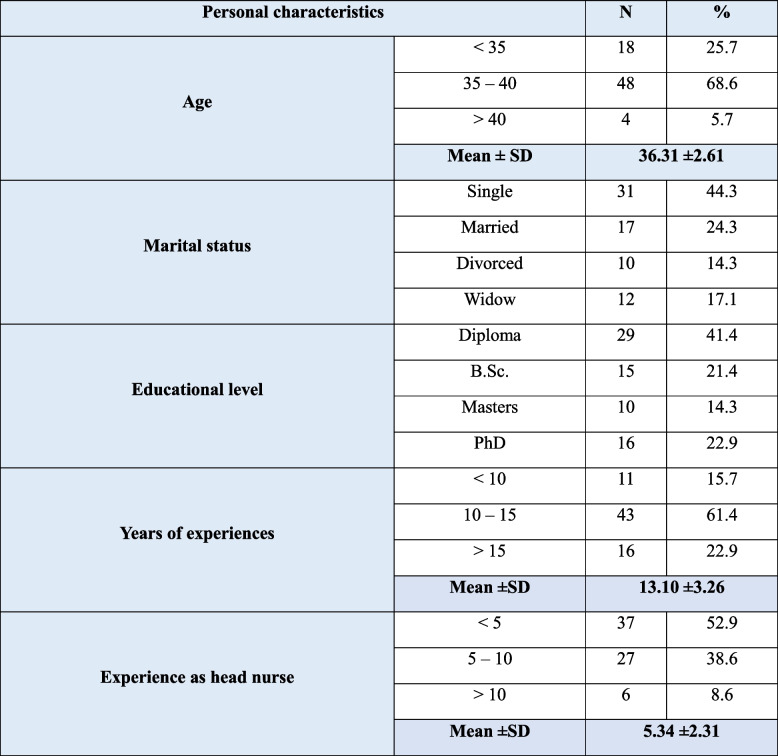
Personal characteristics of studied nurse managers (*n*=70)

As well as, Table 2 illustrates the personal characteristics of the studied nurse staff, indicating that 40.7% of them were aged ranged less than 35 years old with a mean of 38.32 ±4.89, and 66.4% of them were single. Regarding their qualifications, 50.4% of them had a nursing technical institute. Furthermore, 46.9% had from 5 to 10 years of experience with a mean of 9.45 ±2.07.

**Table 2:**
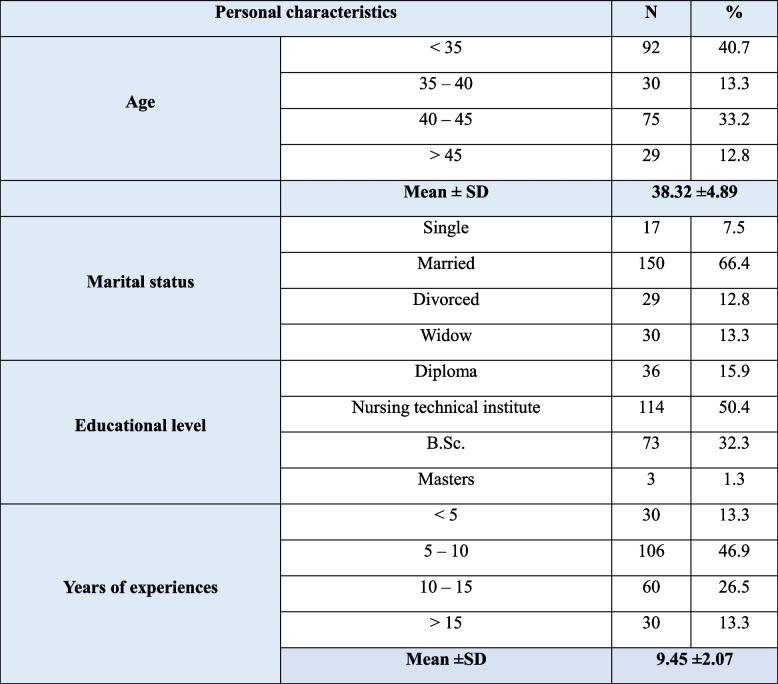
Personal characteristics of studied nurse staff (*n*=226)

### Nurse managers’ knowledge regarding authentic leadership and mindfulness

Table 3 illustrates the studied nurse managers’ knowledge, 67.1% of the studied nurse managers had low knowledge regarding authentic leadership pre-educational program implementation, which improved to 72.9% had good knowledge post-program and slightly decreased to 61.4% after three months of program implementation with statistically significant differences between the program three phases’ scores (pre, post, and follow-up program implementation) (*P* <0.001). In addition, there was a large effect of the educational program on knowledge scores immediately post-program and after 3 months.

**Table 3:**
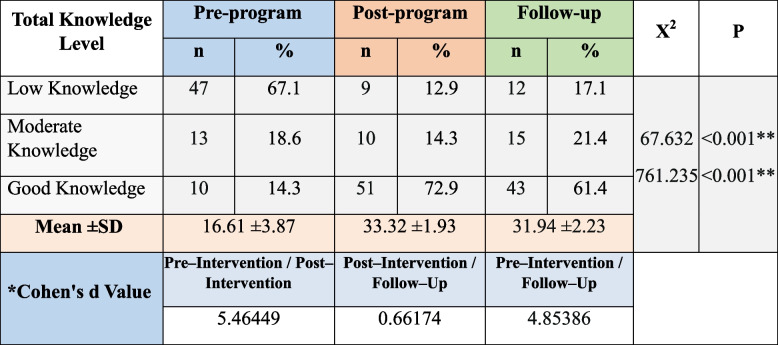
Distribution of the studied nurse managers’ total knowledge pre-, immediate and 3-months post-program (*n* = 70)

^*^Cohen's d: small effect size at <0.5, medium effect size at 0.5< 0.8, large effect size at >0.8

### Nurse managers’ authentic leadership level

Table 4 clarifies the studied nurse managers’ authentic leadership levels, 47.1% of the studied nurse managers had low authentic leadership regarding balanced treatment domain pre implementation of the program which improved to (60%) of them had high level post-program, and (50%) of them had high level after 3 months of implementation the program. Also, 45.7% of them had low authentic leadership regarding the relationship transparency domain pre-implementation of the program which improved to (61.4%) of them had high level post-program, and (45.7%) of them had high level after 3 months of the program implementation. Moreover, there were significant statistical differences between the program three phases’ scores (pre, post, and follow-up program implementation) (*P*<0.001). In addition, as shown by Cohen’s *d* test there was a large effect of the educational program on authentic leadership scores immediately post-program and after 3 months.

**Table 4:**
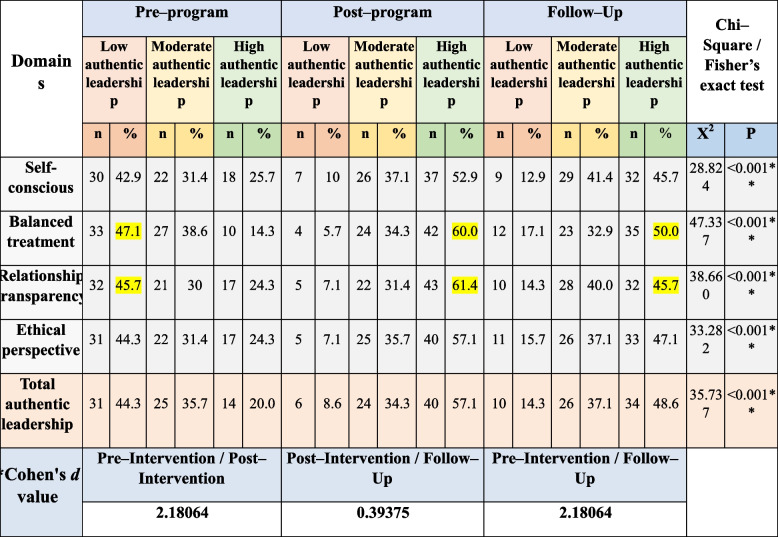
Distribution of the studied nurse managers’ authentic leadership pre-, immediate post-, and after 3 months follow-up (*n* = 70**)**

^*^Cohen's d: small effect size at < 0.5, medium effect size at 0.5 < 0.8, large effect size at > 0.8

### Nurse managers’ mindfulness levels

Table 5 elaborates level of the studied nurse managers’ mindfulness, the studied nurse managers had low mindfulness pre-implementation of the program regarding observation facet (44.3%) and action awareness facet (41.4%), which improved to (71.4%, and 72.9%) had high mindfulness post-program and (54.3%, and 57.1%) after **3** months post-program. Moreover, there were statistically significant differences between the program three phases’ scores (pre, post, and follow-up program implementation) at all domains (P <0.001). In addition, as shown by Cohen’s *d* test there was a large effect of the educational program on mindfulness scores immediately post-program and after 3 months.

**Table 5:**
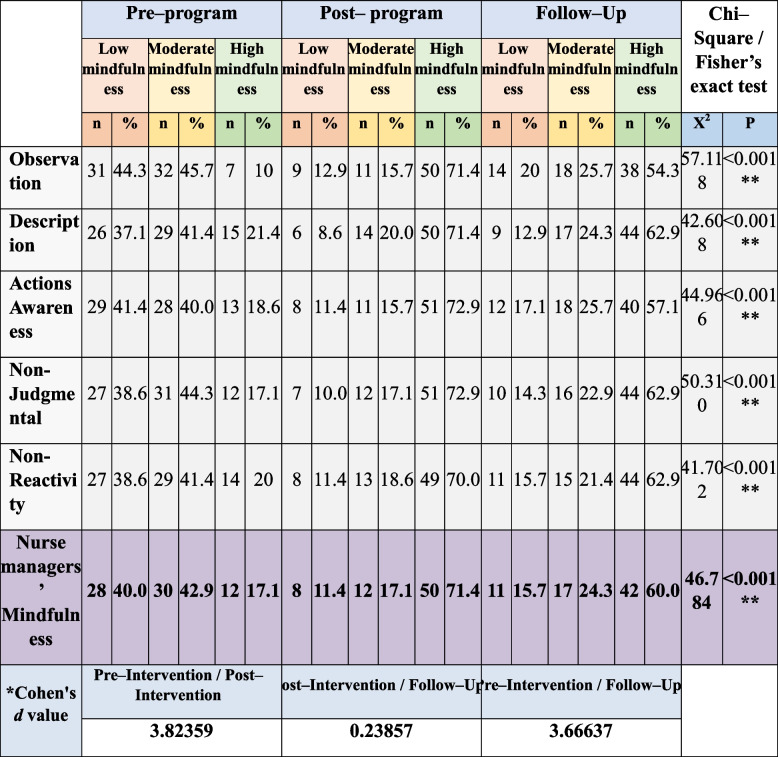
Distribution of the studied nurse managers’ mindfulness pre-, immediate post-, and after 3-months follow-up (*n* = 70)

^*^Cohen's d: small effect size at < 0.5, medium effect size at 0.5 < 0.8, large effect size at > 0.8

### Nurse managers’ managerial competencies as perceived by staff nurses

Table 6 shows the studied nurse manager’ total managerial competencies level, the studied nurse managers had low managerial competencies pre-implementation of the program regarding technical skills (75.2%), conceptual skills (73.9%), and human relationship skills (73%) which improved to (87.2%, 88.5%, 89.8%) had high managerial competencies post-program, and (79.6%, 83.6%, 81.4%) after 3 months post-program. Moreover, there were statistically significant differences between the program three phases’ scores (pre, post, and follow-up program implementation) in the three skills (*P* <0.001). In addition, as shown by Cohen’s *d* test there was large effect of educational program on managerial competencies scores immediately post-program and after 3 months.

**Table 6:**
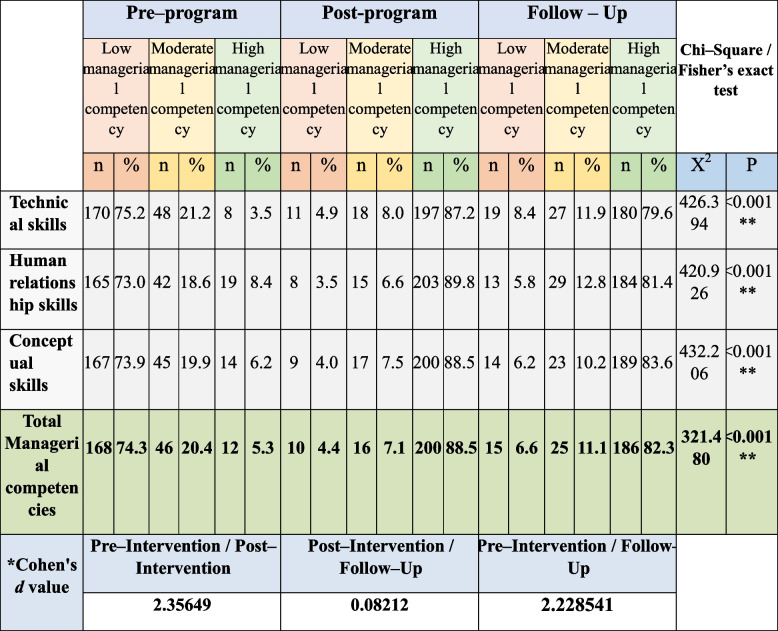
Distribution of the studied nurse managers’ managerial competencies levels as perceived by their nurse staff pre-, immediate post-, and after 3-months (*n* = 226)

^*^Cohen's d: small effect size at < 0.5, medium effect size at 0.5 < 0.8, large effect size at > 0.8

### Relation between study variables

Table 7 revealed that there was a significant statistical relation between the studied nurse managers’ authentic leadership level and their mindfulness and competencies immediately post-program and after three months of implementation the educational program.

**Table 7:**
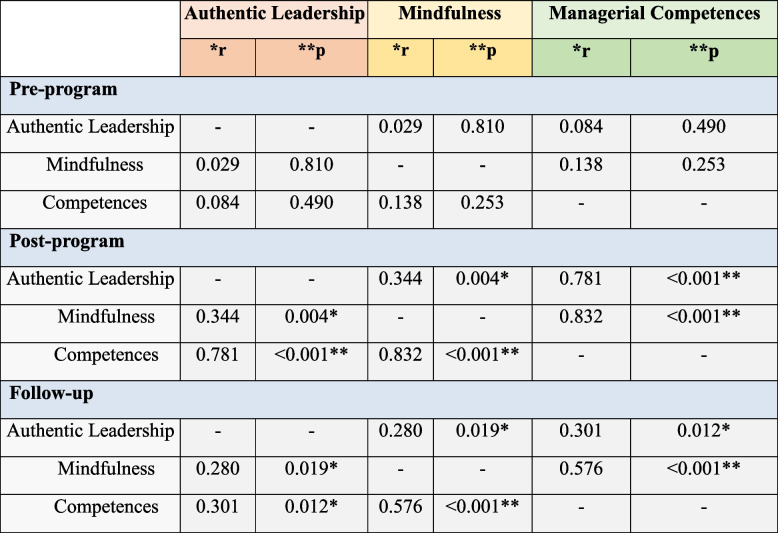
Correlation between nurse managers’ Authentic Leadership, Mindfulness, and Managerial Competence scores

*r: Pearson coefficient ^**^Statistically significant at *p* < 0.05

## Discussion

The current study was directed to explore the effect of an educational program about authentic leadership and mindfulness on nursing managers' competencies in the hospital. According to the present study findings, there was statistically significant positive relation between the educational program with the nurse managers’ scores of authentic leadership, mindfulness, and managerial competencies immediately post-program and after three months of the program implementation. In fact, the program helped those nurse managers in performing their leadership skills effectively, and providing them the opportunity to engage in open conversations, evaluate and value different points of view, share their experiences with others, and learning from others’ stories. According to Elmawla et al. [18] being authentic nurse manager is hard work and takes years of experience in leadership roles. Training program will shorten the period of nurse managers developing authentic leadership by connecting them with truth, creative ideas, journey of self-discovery and sharing experiences. Along the same line, Ali et al. [22] studied the effect of an authentic leadership educational program for head nurses on staff nurses' organizational commitment among 32 nurse managers at Benha University Hospital and found that a statistically significant improvement in the studied head nurses’ mean score regarding their attitude toward authentic leadership thorough program phases. The improvement included the dimensions of self-awareness, internalized moral perspective, balanced processing, relational transparency post-program, and follow-up after the program compared to pre-program scores as reported by head nurses. Also, Dietl and Reb [31] found that a short mindfulness practice (i.e., a 10-minute focused breathing exercise) increased leaders’ felt authenticity, and that leader trait mindfulness was positively related to follower-rated authentic leadership.

Concerning nurse managers’ knowledge regarding authentic leadership and mindfulness**,** the findings of the current study demonstrated that there were highly statistically significant differences between scores of the studied nurse managers’ knowledge regarding authentic leadership and mindfulness in the three study phases. In the pre-program phase, the highest percent of the studied nurse managers had a poor level of authentic leadership which improved to good level immediately post- and after 3 months of the program implementation. This may be due to the most of nurse managers did not attend previous training programs about authentic leadership style and don't recognize the importance of authentic leadership. Although almost all participants' knowledge scores improved after the program was implemented; most head nurses demonstrated a high level of authentic leadership practice. It's possible due to their active involvement and interest in the program sessions and frequent review of their knowledge. As asserted by Ali et al. [22]; and Aboelenein, and Mostafa [23] the training programs regarding authentic leadership were considered a new trend in Egyptian nurses' culture.

This result agreed with Aboelenein, and Mostafa [23] who studied the effect of an educational program about authentic leadership for head nurses on staff nurses' resilience and innovation behavior among 54 nurse managers at the Tanta International Teaching Hospital and found that the studied nurse managers' knowledge and authentic leadership style levels were low pre-program implementation while, they had higher scores with statistically significant differences post-program implementation. Also, Hassan et al. [32] studied the effect of an authentic leadership training program of head nurses on creativity and motivation of nurse staff among 36 nurse managers at Mansoura University hospital and revealed that there were highly statistically significant differences in a total of head nurses about authentic leadership style knowledge at the three times of the program (pre-test, immediately post-, and three months after the program implementation).

Regarding the studied nurse managers’ authentic leadership levels**,** the current study results elaborated that there were statistically significant differences between authentic leadership levels among the studied nurse managers at the three phases of the program. As noticed, the highest percent of nurse managers had low authentic leadership level preprogram regarding self-conscious domain improved to two thirds of them had high level immediately post and half of them had high level after three months of the program implementation. This result explains how the present leadership educational program based on mindfulness effected positively on the studied nurse managers’ self-awareness which increase knowledge of one’s strengths, weaknesses, values, beliefs and emotions, as well as their impact on others [33].

Moreover, the present study findings revealed that there was statistically significant variation in authentic leadership levels regarding domains of balanced treatment of information at the three phases of the program (pre, immediately and, three months post the program implemented), which reflects improvement in the nurse managers’ dealing with others by unbiased openness to differing perspectives on oneself and questioning of one’s positions [8]. This result was supported by Nübold et al. [9] who concluded that by reducing negatively biased cognition, leadership training based on mindfulness may enhance balanced processing of the studied managers. Also, it was observed that there was significant improvement regarding relationship transparency domain, the study findings revealed increased nurse managers’ capacity for displaying empathy and more ability to build more supportive relationships through an improved understanding of others. This result indicates the enhancement in the studied nurse managers’ revealing information, thoughts and sincere emotions to others through engagement in the current program activity. The study conducted by Dietl, & Reb [31] found that managers’ who participated in authentic leadership training based on mindfulness practices were showed increasing in their ethical perspective and relational transparency.

The current study's findings also revealed that there was statistically significant improvement in nurse managers’ ethical perspective scores immediately post and after three months of the program implementation. This result indicted that the educational program increased nurse managers internalized moral perspective, i.e., a process of self-regulation guided by moral values and standards forming the basis for decision making and undertaking action [9]. This finding in the same line with Tan, Peters, & Reb [12] who described that the mindfulness-based leadership training (MBLT) can support leaders in developing clarity regarding their values and purpose in life and enable them to act according to their personal beliefs and truth, supporting ethical perspective.

Regarding nurse managers’ mindfulness levels**,** the present results revealed that the highest percent of the studied nurse managers had low mindfulness pre-implementation of the program regarding observation and action awareness facets, which improved to more than two-thirds of them having high level of mindfulness post-program and more than a half of them had high level after three months post-program. From the researcher’s point of view, these results may be because the mindfulness training program improved nurse managers’ knowledge and provided them with practical methods for enhancing attention and awareness about mindfulness techniques such as breathing and meditation. Actually, they trained for making pausing and paying attention to notice beauty in the environment, transportation, office, and home. Also, most of the studied nurse managers became have the desire to develop themselves rather than pre-program by learning and acquiring new applicable knowledge. Most probably by practicing mindfulness they reduced their stress and enhanced their awareness.

These Findings are consistent with results reported by Elmawla et al. [14] who studied the effect of an educational program about authentic leadership and mindfulness factors on head nurses practice self–evaluation among 70 nurse managers at Elmenshawy General Hospital and Kafer El sheikh General Hospital, and indicated that there was a statistically significant improvement of total practice of mindfulness and each of observing, describing, acting with awareness, nonjudging of inner experience, and nonreactivity to inner experience factors immediate and three months post program than preprogram. And clarifies that, nurse managers’ training in mindfulness practice and meditation techniques improved their ability to focus and concentrate on any task at hand and improved their functioning with subordinates and patients, as well as had a positive effect on their physical and mental workability.

Regarding managerial competencies of the studied nurse managers as perceived by staff nurses as followers**,** teasing apart leaders’ and followers’ perceptions is essential in order to determine whether a behavioral or attributional perspective of authentic leadership is more valid. Thus, in order to fully capture and understand the role of mindfulness for authentic leadership, it would be desirable to consider both followers’ and leaders’ perspectives of authentic leadership simultaneously [9], also, as a positive outcome of the present study, the participation of staff nurses in assessing nurse managers' competencies pre- and post-authentic leadership program indirectly affected nurses’ perceptions of interpersonal justice via authentic leadership.

The present findings indicated that nearly three-quarters of the studied nurse managers had low managerial competencies preprogram in all tested skills as perceived by their staff nurses. The lowest percentage was regarding technical skills followed by conceptual skills. This finding further strengthens the fact that nurse managers in Egypt are not appointed based on clinical proficiencies of each specialty but based on educational certificates only, without testing their technical skills in each department. This would increase conflict and emotional stress during work, which negatively affects nurse managers’ mindfulness. According to Ofei, Paarima, and Barnes [21] technical skills are needed by nurse managers for supportive supervision to ensure that things are done right, and the right thing is done through the transfer of adequate knowledge, skills, and attitude or competencies. Another rationale for these findings may be that they had a higher responsibility and were loaded during the morning shift. However, it might be a lack of confidence in their ability and a lack of self-belief. Also, these results were supported by Abd-Elmoghith, and Abd-Elhady [34] Who assessed nurse managers' competencies and their relation to their leadership styles among 65 nurse managers at the Oncology Center -Mansoura University, and revealed that slightly less than half of nursing managers had a low level of competency.

The results of the current study indicated there were statistically significant variations in nurse managers’ managerial competencies scores regarding human relationship skills as perceived by staff nurses in the three study phases. It was noticed that preprogram the most of staff nurses reported that the nurse manager has small extent the ability to market care and urges teamwork, which improved post-program and after three months of the program to the most of them reported that the nurse director has large extent the ability to market care and urges teamwork. In this context, authentic leadership can serve as a positive role model for subordinates in the workplace. Before making a decision, authentic leaders prefer to encourage employees' independent thinking and expressive behavior. On the other hand, authentic leadership fosters trust and personal support among employees, boosting self-efficacy and meeting competency requirements [35]. In the same direction with the current results Mashavira, & Chipunza [36] who found that there was strongly positive effect of authentic leadership training on managers’ skills regarding building constructive relation with their subordinate especially improve teamwork and supporting their self-efficacy.

Furthermore, the current results presented that there were statistically significant differences in nurse managers’ scores regarding conceptual skills as perceived by staff nurses at the study three phases. As evidence, pre-program about three quarters of staff nurses reported that the nurse manager has small extent the ability to analyze effectively and plan strategically, which improved post and after three months of the program to the most of them reported that the nurse director has large extent the ability to analyze effectively and plan strategically. These findings agreed with Gunawan et al. [37] who concluded that there was statistically significant improvement in the managers conceptual competencies after refining of their managerial core competencies through implementation of authentic leadership training.

Relation between study variables**,** the present results there was a significant statistical relation between the studied nurse managers’ authentic leadership level and their mindfulness and competencies immediately post-program and after three months of implementation the educational program. This result can be positively extended to nurses’ work attitudes via authentic leadership. Along the same line, Elmawla et al. [18] found a positive relation between nurse managers’ mindfulness and their knowledge, while there was a positive relation non-significant relation between nurse managers’ authentic leadership and their knowledge. Also, Nübold et al. [9] confirmed a positive relation between leaders’ trait mindfulness and authentic leadership as rated by both followers and leaders, and showed that the intervention increased authentic leadership via gains in leaders’ mindfulness.

## Conclusion

### Based on the findings of the present study, it can be concluded that

The educational program about authentic leadership and mindfulness was effective in enhancing nurse managers’ managerial competencies. Through the evaluation of nurse managers immediately post the program and after 3 months follow-up, it was found that the participants showed a good level of knowledge and attitude regarding authentic leadership, such as relationship transparency and balanced treatment. also, they experienced high perception regarding mindfulness practices and the effect of these practices on their managerial competencies, especially conceptual skills. The study recommended conducting periodical training programs about authentic leadership to update nurse managers’ essential knowledge. And periodically training regarding mindfulness practices to increase nurse managers’ observing, describing, acting with awareness, non-judging of inner experience and non-reactivity to inner experience to reduce work stress and turnover.

### Implications for practice and future directions

The findings provide multiple fields of study relating to nursing leadership development that may benefit from a better understanding of how to improve nurse managers’ training through the addition of mindfulness practice. The studied nurse managers and staff nurses reported improved workplace interaction, also linking mindfulness practice with more prosocial behavior especially increased attentive listening and more response to others. The study findings also revealed increased nurse managers’ capacity for displaying empathy and more ability to build more supportive relationships through an improved understanding of others. The researcher recommends further research examining the effect of that training programs based on mindfulness and authentic leadership on nurses’ productivity and empowerment of nurses in health organizations. As well as, it recommended to develop various trainings and workshop about authentic leadership for whom in a nursing managerial position to increase their knowledge and competencies regarding their authentic leadership. The researcher also recommends further research in healthcare organizations to utilize the findings regarding authentic leadership and mindfulness and implications of this study to determine ways to overcome work-life balance issues that cause undue stress to nurse managers in various leadership roles. This will allow nurse managers to advance into more senior-level or higher leadership roles while providing them with the confidence and abilities to manage stress knowing that their career trajectories are attainable.

### Limitations of the study

There are certain limitations to this study. First, the rating of knowledge and attitude toward authentic leadership were measured by nurse managers themselves; hence the responses of nurse managers can be overvalued as a result of a tendency of them to report what a researcher expects to hear, and/or what may reflect positively on them. Second, it was not possible to use randomization or a control group in this study, and this issue can be affected by personal bias. To address this issue, several procedural remedies were applied by improving the scale items’ clarity by pilot testing with nurses who had the same inclusion criteria. Also, the participation of the staff nurses in the observation of the effect of the educational program on nurse managers’ competencies was to overcome the concern of bias.

## Data Availability

Due to confidentiality concerns, the data and materials used in the current study cannot be made publicly available. However, they are available from the corresponding author upon reasonable request.
